# Therapeutic community-oriented day treatment program for Korean women with alcohol use disorder: a non-randomized pilot feasibility trial

**DOI:** 10.1186/s13722-022-00297-3

**Published:** 2022-02-22

**Authors:** Insuk Lee, Mihyoung Lee, Scott Seung W. Choi

**Affiliations:** Department of Nursing, College of Medicine, Inha University, 100 Inha-ro, Michuhol-gu, Incheon, 22212 Republic of Korea

**Keywords:** Therapeutic community, Alcohol use disorder, Women, Day treatment, Alcohol abstinence self-efficacy, Forgiveness, Spirituality

## Abstract

**Background:**

The prevalence of alcohol use disorder (AUD) among women in South Korea has been rising, causing public health problems. Yet women’s treatment needs are mostly unmet in South Korea due to the lack of women-focused treatment programs. This study evaluated the feasibility, acceptability, and clinical outcomes of a therapeutic community (TC)-oriented day treatment program for Korean women with AUD on alcohol abstinence self-efficacy, forgiveness, and spirituality.

**Methods:**

The current study employed a quasi-experimental, non-equivalent control group design with a pretest and posttest. Participants were assigned to 6-month TC-oriented day treatment program (n = 19) or usual treatment (n = 21). Feasibility (treatment completion rate) and acceptability (overall program satisfaction) were assessed. Alcohol abstinence was measured as a clinical outcome at baseline, 3 months, and 6 months. Repeated measures using Alcohol Abstinence Self-Efficacy (AASE), Enright Forgiveness Inventory-Korea (EFI-K), and Spiritual Assessment Scale (SAS) were also obtained from both conditions at those three time points.

**Results:**

Fifteen participants (78.9%) in the intervention group successfully completed the program. The overall program satisfaction ratings were very high (4.9 ± 0.2). Continuous abstinence rates at 6 months were significantly higher in the treatment group (78.9%) than in the control group (9.5%). Results of the two-way repeated measures ANOVA indicated that statistically significant two-way (group × time) interaction effects were found for the intervention group on AASE, EFI-K, and SAS but not for the control group on any of the outcomes.

**Conclusion:**

The current study demonstrated the feasibility and acceptability of implementing a TC-oriented intensive day treatment program to promote recovery in Korean women with AUD. This intervention merits further investigation as a potential strategy to help address alcohol abstinence self-efficacy, forgiveness and spirituality.

*Trial registration*: KCT0006386 (Cris.nih.go.kr).

**Supplementary Information:**

The online version contains supplementary material available at 10.1186/s13722-022-00297-3.

## Introduction

The harmful use of alcohol is high on the global health agenda. Three million deaths worldwide are attributable to the consumption of alcohol every year [1]. It is also a major public health issue in South Korea. Reinforced by social acceptability of drunkenness, the rates of heavy drinking and alcohol use disorder are especially high in South Korea compared to other Organisation for Economic Co-operation and Development (OECD) countries [2, 3]. The emerging trends of the past five years indicate that the prevalence of alcohol use disorder (AUD) for women in South Korea has been increasing by 1.60% every year whereas it has been declining for men by 1.73% annually [4].

Among the challenges in preventing and reducing alcohol-related harms in South Korea is the lack of resources to finance treatment programs. There are only seven specialist hospitals in the entire country providing inpatient treatment services to individuals with AUD which use almost all of the government budget appropriated for supporting treatment programs [2]. Kim et al. [5] suggest that more funds should be spent on establishing outpatient treatment programs as part of an integrative treatment system to increase treatment utilization among individuals with alcohol use disorder.

Therapeutic communities (TCs) are a form of long-term residential treatment for individuals with substance use disorder (SUD) [6]. From its inception in the 1950s to the present day, TCs have demonstrated effectiveness in achieving positive outcomes for substance use, criminality, and employment [7, 8]. The underlying notion of TCs is that SUD is a disorder of the ‘whole person,’ of which recovery requires lifestyle changes extending beyond a simple abstinence from substance use [7]. To facilitate these global changes in lifestyle, a unique treatment approach called ‘community as method’ is employed which can be defined as ‘the purposive use of the community to teach individuals to use the community to change themselves.’ [9].

In response to the increased need for treatment programs targeting populations with special needs, TCs have been modified during the past five decades in terms of setting, curriculum, length of stay, and modality [7, 10]. One population that TCs have adjusted their programs to meet the needs of was women [6]. Numerous studies have reported gender differences among individuals with SUD in such aspects as patterns of substance use, physical and sexual abuse histories, personal responsibility, psychological symptoms, and barriers to treatment. As a result, researchers have recognized the need for TC programs specifically designed to meet the complex needs of women with SUD [11–14]. For example, for those women who cannot stay overnight at a residential facility due to their family and professional responsibilities or obligations, a modified residential TC program that offers “day treatment” can be a flexible treatment option. In fact, some Korean women with AUD cited the residential nature of treatment and medical services as one of the barriers to seeking treatment [15]. Other services that women may need can include medical and mental health services, trauma-informed approach, parenting education, individual/family counseling, and case management [12, 16].

Research shows that modified TCs, regardless of treatment modality and structure, are, overall, effective in improving treatment outcomes [7, 8]. However, most studies focus on reporting objective outcomes concerning substance use such as retention rates, length of stay in treatment, recidivism rates, and employment status [8]. These studies have largely neglected subjective outcome measures [8, 17]. Consequently, we have little information about the treatment effect on psychological outcomes that may be important measures of recovery from the TC’s ‘whole person’ perspective.

Among the subjective outcome indicators related to recovery are alcohol abstinence self-efficacy, forgiveness, and spirituality. Alcohol abstinence self-efficacy is a crucial element in recovery from alcohol dependence [18]. There is considerable empirical evidence that abstinence self-efficacy can predict abstinence in people with SUD [19, 20]. Forgiveness, which can be defined as the willful abandoning of resentment and related responses [21], has been recognized as a critical concept in the treatment of SUD. Aspects of forgiveness can be identified in almost all validated forms of treatment and psychotherapies for alcohol problems (e.g., 12-Step Model of addiction and recovery, Motivational Enhancement Therapy, etc.) [22]. Yet, very little empirical investigation has been conducted in examining the relationships between forgiveness and alcohol-related outcomes. As with forgiveness, spirituality has also been argued to be highly relevant to the process of recovery from SUD, most notably in faith-based treatment programs such as the 12-Step Model of Alcoholics Anonymous [23]. Empirical evidence suggests that spirituality may be a protective factor from SUD [24] as well as a predictor of post-treatment abstinence [25]. Despite its importance in recovery from SUD, spirituality has not been examined as often in substance treatment studies as other variables, such as self-efficacy [26].

Despite a number of studies demonstrating the efficacy of TCs for a variety of populations with special needs, few TCs have been introduced to the substance use services in South Korea [27]. At present, there is only one residential TC program that caters to women with AUD in South Korea [28]. Given the increasing number of women with AUD and insufficient access to treatment services for women in Korea, development and evaluation of a TC-oriented outpatient treatment program designed to meet the complex needs of women is warranted.

Therefore, we developed a community-based intensive day treatment program based on the TC model, which can be applicable to women with AUD living in South Korea. The primary aims of this study were to evaluate the feasibility and acceptability of adapting a traditional TC treatment for Korean women with AUD in a day treatment setting. As secondary aims, the study examined the effects of the treatment on alcohol abstinence, alcohol abstinence self-efficacy, forgiveness, and spirituality. We focused on alcohol abstinence rather than alcohol moderation because TCs have traditionally provided abstinence-based treatment services. We anticipated that the day treatment program would be feasible to be implemented in the community and acceptable among Korean women with AUD. We also expected participants with the treatment to have higher alcohol abstinence rates and higher levels of alcohol abstinence self-efficacy, forgiveness, and spirituality compared to those without the treatment.

## Methods

### Study design

This pilot study employed a quasi-experimental, non-equivalent control group design with a pretest and posttest. Allocation was not randomized. Instead, after being determined eligible, participants were assigned to treatment groups in consultation with the research staff. Participants had the flexibility to choose the appropriate group if they had specific needs or responsibilities (e.g., employment, family obligations) that might hinder their participation in the trial. Participants in the intervention arm were admitted to a 6-month day treatment program while participants in the control arm received the usual treatment services for the same duration. All procedures were approved by the Inha University Institutional Review Board (171009-1AR), and written informed consent was obtained from all participants.

### Participants and setting

Participants were recruited using referrals by local psychiatrists and flyers posted at drug and alcohol treatment centers in Suwon, the capital city of South Korea’s most populous province. Screening interviews were conducted over the phone by research staff. Participants were enrolled on a rolling basis from November 2017 to April 2020.

Eligible participants were women aged 19 years and older, diagnosed of AUD by a physician, having a lifetime history of medical treatment for AUD, and willing to commit to the study protocol. Participants were excluded from the study if they were at risk for suicide or homicide, unable to provide informed consent due to cognitive impairments, or had medical or psychiatric illnesses that would adversely affect their study participation. Participants received no monetary incentives or compensation.

The study was conducted in the Suwon Community Addiction Management Center in Gyeonggi Province, South Korea, a non-profit community-based agency and affiliate of the Inha University College of Medicine. This agency has historically operated multiple treatment programs for alcohol use disorder, substance use disorder, gambling addiction, and internet addiction.

### Intervention

The day treatment program was the modified TC adapted to women. The program director and the program consultant had received comprehensive training in the operation of a residential therapeutic community at the Daytop International in 2004 and have since been involved in numerous projects practicing the TC model in South Korea.

The modified TC was similar to the traditional TC in terms of structure, processes, and interventions. However, modifications were made to the TC approach to meet the needs of women with AUD and make it more acceptable to the cultural setting. For example, confrontation technique was not used as much in our program as would have been in a traditional TC for fear that it would be considered unacceptable or cause unnecessary stress in Korean women [29]. Furthermore, empowerment and mutual respect, rather than authoritarianism, were prioritized at all levels of interaction to avoid possible retraumatization in women who may have had experienced physical and/or sexual abuse [10]. In sum, the day treatment program was different from the standard TC in that it was more flexible (e.g., fewer activities, shorter interactions), less intense (e.g., less confrontation, fewer sanctions), and more sensitive to individual differences (e.g., more explicit affirmation of individual achievements).

**Fig. 1:**
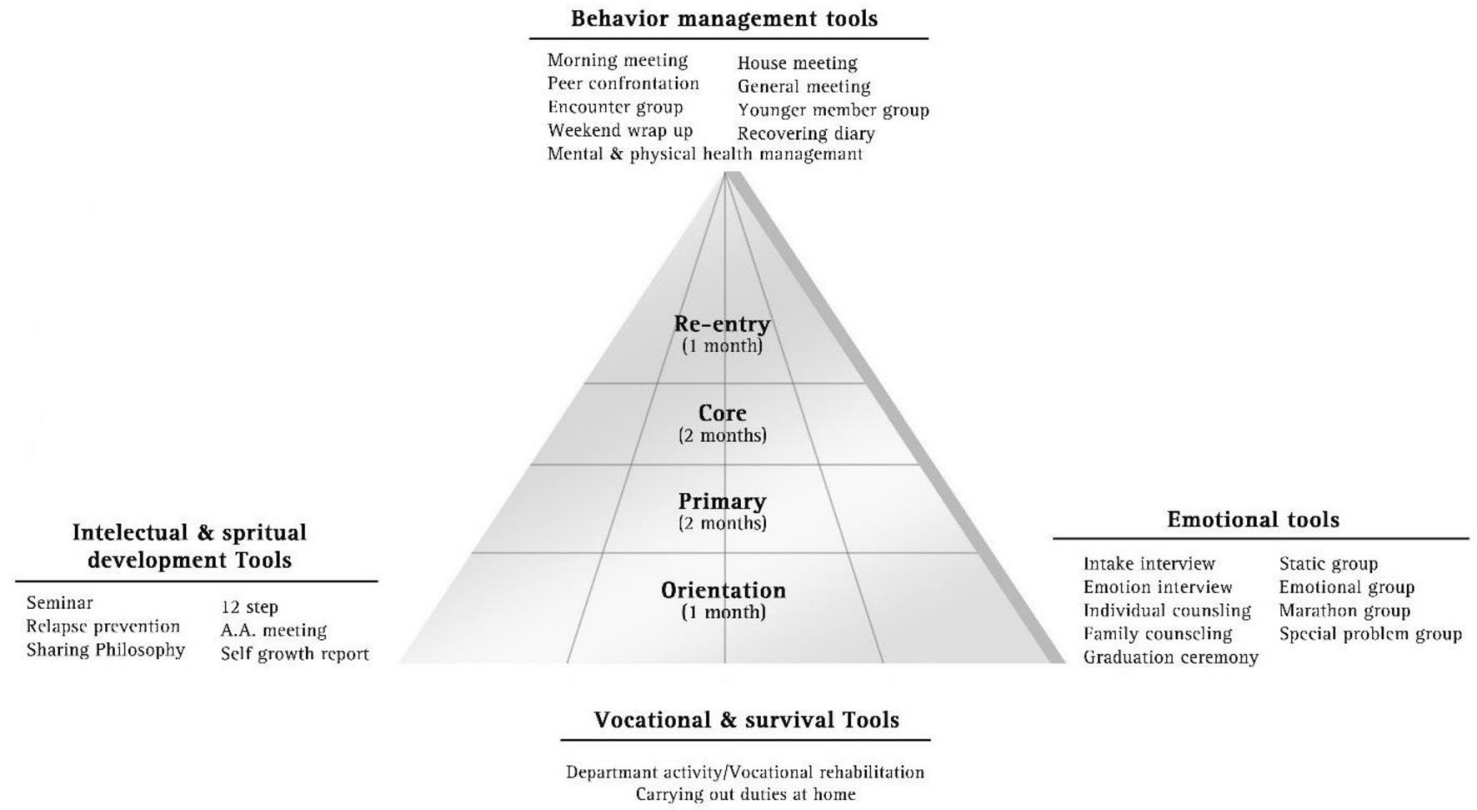
The categories of the treatment activities used in the day treatment program. Note. The figure shows the fundamental components and the treatment activities of the day treatment program. Adapted from Application of Therapeutic Community in the Rehabilitation of Addicts, [42] by M. Lee, S. Choi, B. Chung, and I. Lee, 2005, Hinson Publishing Co. Copyright 2005 by Mahjong Lee. Adapted with permission

Despite these modifications, the essential elements of the standard TC were retained in the program such as the ‘community as method’ approach, the mutual ‘self-help’ principle, and the ‘right living’ concept. The day treatment program operated 3 days per week from 9 am to 5 pm. The highly structured daily regimen included morning/evening meetings, mindfulness meditation, education groups and peer seminars (see Table 1). The treatment activities are organized into four categories (i.e., behavior management, intellectual/spiritual, emotional, vocational-survival skills), as outlined in Fig. 1.

**Table 1:** Overview of the day treatment model for women with alcohol use disorder

Item	Description
Model	Therapeutic community
Modality	Intensive day treatment
Goal	Recovery from alcohol use disorder
Duration	6 months (3 days/week)
Staff	Program director (registered nurse), associate director (registered nurse), counselor (licensed clinical social worker), and 2 ex-addict role models (graduates from a residential TC program)
Methods	Morning/evening meeting, house meeting, education groups, encounter groups, and peer seminars
Gender-specific groups	Parenting education, vocational training
Sanctions	There are two cardinal rules which, if violated, would lead to expulsion from the program: No alcohol use (alcohol breath testing is required prior to commencing a daily regimen. The very first violation will lead to expulsion from the program.) No absence from the program (two or more unexcused absences at each phase will lead to expulsion from the program.) Other disciplinary sanctions determined by the community may be imposed in response to breaches of the community standards.
Stages	Phase I (first 1 month): orientation and induction Phase II (months 1–3): primary treatment Phase III (months 3–5): transition phase Phase IV (months 5–6): re-entry phase

Outside of the official program hours, participants were expected to attend other support group meetings (e.g., 12-step programs) in keeping with the TC culture. On weekends, participants would engage in recreational and leisure activities at off-site locations. These additional activities were meant to prolong the influence of the TC. Additionally, medical and psychiatric services were provided to all participants on a weekly basis at affiliated clinics.

### Control

Control group participants received ‘treatment-as-usual’ which was the case management services provided by a social worker. A social worker specializing in substance use disorders from the addiction management center made monthly home visits during which she assessed clients’ conditions, assisted with their immediate problems, monitored evidence of alcohol use, offered counseling on addiction management, and made referrals as needed to the appropriate community resources. No structured psychosocial intervention was routinely provided to the control group.

### Measures

#### Primary outcomes

Feasibility was determined by assessing the percentage of participants who remained continuously in treatment from baseline to study completion while acceptability was assessed by examining the post-intervention survey asking participants to rate overall satisfaction with the program on a 5-point scale (very dissatisfied to very satisfied) and their willingness to recommend on a 5-point scale (not likely to very likely). The predetermined thresholds for feasibility and acceptability were an overall program completion rate of 50% and a mean score of 4.0 on the two post-intervention survey items, respectively.

#### Secondary outcomes

The secondary outcomes were rates of alcohol abstinence from baseline to study completion and three psychological outcome measures related to recovery (i.e., alcohol abstinence self-efficacy, forgiveness, and spirituality). These measures were assessed at three time points: pre-intervention (baseline), mid-intervention (3 months), and post-intervention (6 months). The proportion of the participants abstinent from alcohol in each treatment condition were calculated at each time point. Abstinence at baseline was assessed based on each participant’s self-report of past month use. Abstinence during the following periods was assessed by breath alcohol tests at the treatment center (3 days per week) for the intervention group and monthly self-reports for the control group. Participants were considered abstinent if all test results or self-reports without any missed test were negative for alcohol use. Intention-to-treat analysis was used which counted any drop-out or loss to follow-up as relapse.

Using the Alcohol Abstinence Self-Efficacy scale (AASE) [30], the participant’s efficacy in abstaining from alcohol in 20 high-risk situations was assessed on a 5-point Likert scale (0 = not at all to 4 = extremely). A total score was derived from the sum of 20 items (range 0–80). Higher scores indicate higher self-efficacy to abstain from drinking. For this study, a validated Korean translation of AASE [31] was used (alpha = 0.95).

Forgiveness was measured using the validated Korean version of the Enright Forgiveness Inventory (EFI-K) [32, 33]. Participants rated the extent to which they would forgive an offender to improve their mental health and well-being on a 6-point Likert scale (1 = strongly disagree to 6 = strongly agree) across 30 items (range 30–180). Higher scores indicate higher level of forgiveness (alpha = 0.91).

Spirituality was measured using the 28-item Korean version of the Howden’s Spiritual Assessment Scale (SAS) [34]. Participants rated the questions pertaining to the critical attributes of spirituality on a 6-point Likert scale (1 = strongly disagree to 6 = strongly agree). Scores range from 28 to 168 with higher scores indicating a higher degree of spirituality (alpha = 0.94).

### Data analysis

Although a statistical test of efficacy was not the primary objective of this pilot trial, we estimated a sample size using a previous study as a reference point. The former study examined the effectiveness of a TC intervention for imprisoned Korean men with SUD using the alcohol abstinence self-efficacy scale [27]. A target sample size of 18 participants in each trial arm was calculated for repeated measures ANOVA to detect a large effect (Cohen’s *f* = 0.41) using G*Power 3.1 with 80% power at a 5% significance and 20% attrition assumed. This sample size also complies with recommendations for designing a pilot study [35].

First, background characteristics and outcome variables were compared between the two conditions using Fishers’ exact tests (or Chi-square tests) for categorical variables and independent samples *t*-tests for continuous variables to detect any between-group baseline differences. Descriptive statistics were used to assess feasibility and acceptability. Cochran’s Q test was used to examine differences in the proportions of participants who abstained from alcohol over the course of the study. Additionally, pairwise McNemar tests with the Bonferroni alpha adjustment were used to find significant differences between paired proportions in each condition.

Two-way repeated measures ANOVA analyses were conducted to analyze treatment outcomes (i.e., change over time by intervention condition) using the intention-to-treat principle with the last observation carried forward (LOCF) approach to impute any missing values. Sphericity, which is one of the required assumptions for repeated measures ANOVA, was evaluated by Mauchly’s test of sphericity. In cases where Mauchly’s sphericity test was significant (i.e., the sphericity assumption not met), the Greenhouse–Geisser correction was used to produce a valid *F*-ratio.

Separate repeated measures ANOVAs were run for each of the three outcome variables: self-efficacy, forgiveness, and spirituality. Each model included a variable representing time (pre-intervention, mid-intervention, post-intervention), group membership (intervention or control), and the time × group interaction. To adjust for potential confounding factors, variables that were significantly different between the two conditions at baseline were added as covariates. Effect sizes were measured as partial eta-squared (η^2^). Lastly, we performed pairwise comparisons to determine the pair of time points where there was a significant difference. All analyses were performed using SPSS Statistics version 25.

## Results

### Demographic and background characteristics

**Fig. 2:**
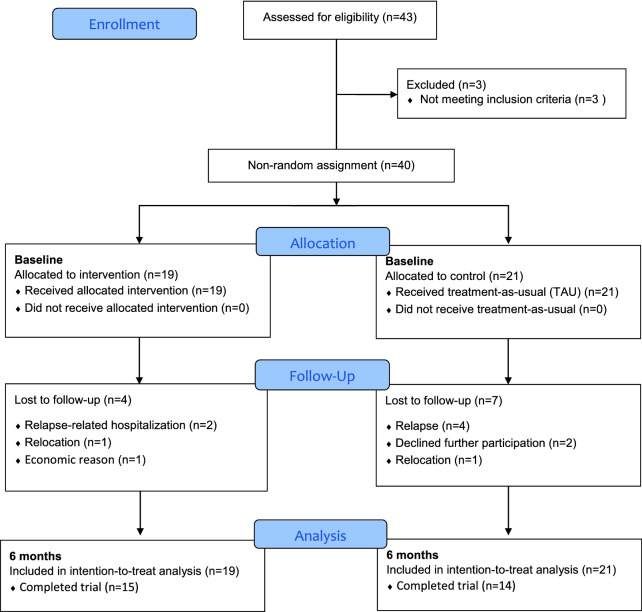
Consort flowchart

As shown in Fig. 2, a total of 40 women met all eligibility criteria, and 19 women were assigned to the intervention group and 21 to the control group. Of these 21 women, 9 were assigned to the control group because they preferred the control condition to the intervention due to their personal reasons: 5 were working full-time and could not leave to attend treatment, 2 had children to take care of at home, 1 cited reasons of household and family responsibilities, and 1 reported transportation challenges. Baseline participant characteristics are summarized in Table 2: 45% of participants were married or living together; 43% were religious; 85% had a family history of AUD; 23% were unemployed; and 75% reported drinking alcohol in the past month. The mean (SD) age was 49.2 (11.9) years (see Additional file 1 for more background information).

**Table 2:** Background characteristics and outcome variables of participants at baseline by treatment condition (intention-to-treat sample)

Variable	Total (n = 40)	Intervention (n = 19)	Control (n = 21)	χ^2^ or *t* test
	n (%) or Mean ± SD	n (%) or Mean ± SD	n (%) or Mean ± SD	*p* value
Age (year)	49.2 ± 11.86	51.1 ± 10.56	47.5 ± 13.21	0.347
Marital status				0.313^a^
Married/living together	18 (45.0%)	11 (57.9%)	7 (33.3%)	
Separated/divorced/widowed	15 (37.5%)	6 (31.6%)	9 (42.9%)	
Never married	7 (17.5%)	2 (10.5%)	5 (23.8%)	
Religious affiliation				0.061
Yes	17 (42.5%)	11 (57.9%)	6 (28.6%)	
No	23 (57.5%)	8 (42.1%)	15 (71.4%)	
Subjective socioeconomic status				1.000^a^
High-to-middle	1 (2.5%)	0 (0%)	1 (4.8%)	
Middle	14 (35.0%)	7 (36.8%)	7 (33.3%)	
Middle-to-low	25 (62.5%)	12 (63.2%)	13 (61.9%)	
Trauma				
Lifetime physical trauma/abuse	28 (70.0%)	14 (73.7%)	14 (66.7%)	0.629
Lifetime sexual trauma/abuse	8 (20.0%)	5 (26.3%)	3 (14.3%)	0.442^a^
Family history of alcohol use disorder				1.000^a^
Yes	34 (85.0%)	16 (84.2%)	18 (85.7%)	
No	6 (15.5%)	3 (15.8%)	3 (14.3%)	
Number of previous hospitalizations for alcohol use disorder				0.413^a^
0	7 (17.5%)	2 (10.5%)	5 (23.8%)	
≤ 2	14 (35.0%)	6 (31.6%)	8 (38.1%)	
≥ 3	19 (47.5%)	11 (57.9%)	8 (38.1%)	
History of suicide attempt				0.664^a^
Yes	34 (85.0%)	17 (89.5%)	17 (81.0%)	
No	6 (15.0%)	2 (10.5%)	4 (19.0%)	
Employment status				0.457^a^
Employed	9 (22.5%)	3 (15.8%)	6 (28.6%)	
Unemployed	31 (77.5%)	16 (84.2%)	15 (71.4%)	
Outcome variables				
Alcohol use in the past month	30 (75.0%)	13 (68.4%)	16 (76.2%)	0.583
AASE	38.10 ± 16.13	31.00 ± 12.51	44.52 ± 16.59	0.006**
EFI-K	73.07 ± 26.03	70.53 ± 28.49	75.38 ± 24.05	0.563
SAS	75.43 ± 20.13	66.05 ± 13.44	83.90 ± 21.65	0.004**

*AASE* alcohol abstinence self-efficacy, *EFI-K* Enright Forgiveness Inventory-Korea, *SAS* Spirituality Assessment Scale

^a^Fisher’s exact test; **p < 0.01

Pearson’s Chi-squared test and independent t-tests indicated that there was no significant difference between the intervention and control groups in demographic characteristics at baseline. However, significant differences were observed for alcohol abstinence self-efficacy and spirituality between the two groups at baseline. These variables were controlled for in the two-way ANOVA with repeated measures.

### Treatment feasibility and acceptability

Of 19 participants who received the intervention, 15 (79%) completed the program and 4 (21%) dropped out. Reported reasons for drop-outs were relapse (n = 2), relocation (n = 1), and economic issue (n = 1). Of 21 participants in the control group, 7 (33%) were lost to follow-up for undisclosed reasons. The difference in the proportions of drop-outs between the two groups was not statistically significant (*z* = − 0.869, *p* = 0.384). As for the evaluation of acceptability, the post-intervention program satisfaction ratings (scored on 1–5 Likert scale) were very high (4.9 ± 0.2). Participants particularly scored the highest rating for ‘willingness to recommend the program to others’ (5.0 ± 0.0). No adverse events were reported.

### Treatment effect: alcohol abstinence

At baseline, Chi-square analysis showed no significant differences in the proportions of abstinent participants between the intervention and control groups (*χ*^*2*^ = 0.302, *p* = 0.583). Table 3 shows that of 19 participants in the intervention group, 15 (78.9%) remained abstinent for 6 months. In contrast, only 2 (9.5%) of 21 participants in the control group reported complete abstinence over the same period. While the proportions of participants abstaining from alcohol in the intervention group were not affected by time (*Q*^2^ = 1.75, *p* = 0.417), the proportions of participants reporting abstinence in the control group dropped significantly over 6 months (*Q*^2^ = 20.38, *p* < 0.001): baseline (76.2%), 3 months (23.8%), 6 months (9.5%). An exact McNemar’s test further indicated that a statistically significant change in the relapse rates had occurred between baseline and 3 months in the control group (N = 21, Exact *p* = 0.003).

**Table 3:** Changes in proportion of alcohol abstinence vs. relapse among participants by study condition over 6 months

		Baseline (T0)	3 months (T1)	6 months (T2)	Cochran's* Q* test *(df* = *2)*^d^	Pairwise McNemar tests
		Pre-intervention	Mid-intervention	Post-intervention		
		n (%)	n (%)	n (%)		
Intervention	Abstinence	13 (68.4%)^a^	16 (84.2%)^b^	15 (78.9%)^b^		T0 and T1, *p* = 0.453
	Relapse	6 (31.6%)^a^	3 (15.8%)^b^	4 (21.1%)^b^	*Q* = 1.75, *p* = 0.417	T1 and T2, *p* = 1.000
	Total	19 (100%)	19 (100%)	19 (100%)		T0 and T2, *p* = 0.727
Control	Abstinence	16 (76.2%)^a^	5 (23.8%)^a^	2 (9.5%)^a^		T0 and T1, *p* = 0.003*
	Relapse	5 (23.8%)^a^	16 (76.2%)^a^	19 (90.5%)^a^	***Q* = 20.38, *p* < 0.001**	T1 and T2, *p* = 0.375
	Total	21 (100%)	21 (100%)	21 (100%)		T0 and T2, *p* < 0.001*

Bold values denote statistically significant results

All participants lost to follow-up were treated as relapses per intention-to-treat principle

Cochran’s Q tested the null hypothesis that the proportions of ‘relapse’ were the same over time

^*^McNemar Exact *p* < 0.017 (Bonferroni correction)

^a^Based on participant self-report

^b^Based on breath alcohol test

### Treatment effect: alcohol abstinence self-efficacy, forgiveness, and spirituality

**Fig. 3:**
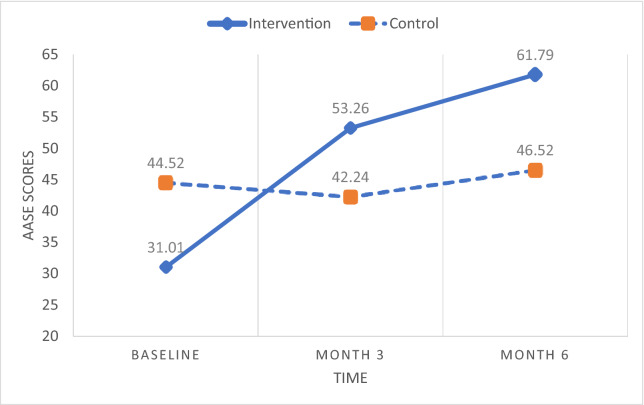
Abstinence self-efficacy scores over time by group. *AASE* alcohol abstinence self-efficacy

**Fig. 4:**
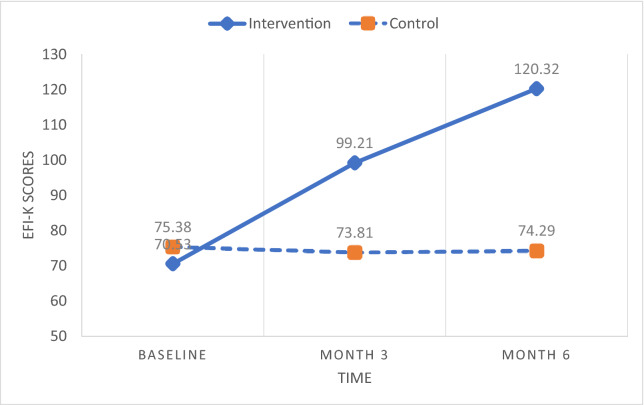
Forgiveness scores over time by group. *EFI-K* Enright Forgiveness Inventory-Korea

**Fig. 5:**
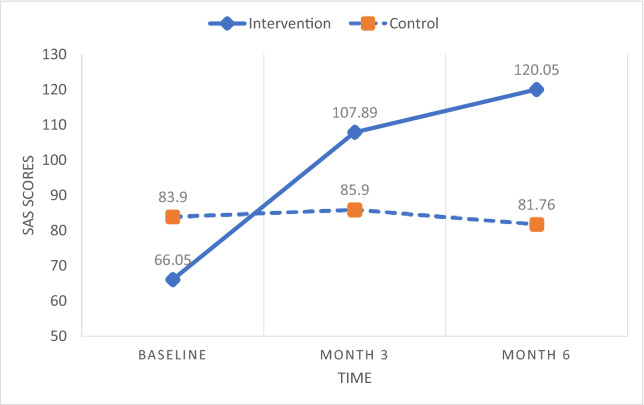
Spirituality scores over time by group. *SAS* Spirituality Assessment Scale

Two-way repeated measures ANOVA examined the differences between the two groups over time in alcohol abstinence self-efficacy, forgiveness, and spirituality (Table 4). Mauchly’s test of sphericity indicated that the assumption of sphericity was met for abstinence self-efficacy whereas it was violated for forgiveness (ε = 0.731) and spirituality (ε = 0.733). Thus, the Greenhouse–Geisser correction was used to correct for this bias for forgiveness and spirituality. There were statistically significant two-way (i.e., group × time) interaction effects on abstinence self-efficacy [*F* (2, 74) = 23.77, *p* < 0.001, η^2^ = 0.391], forgiveness [*F* (1.39, 52.74) = 46.16, *p* < 0.001, η^2^ = 0.548], and spirituality [*F* (1.36, 50.21) = 39.65, *p* < 0.001, η^2^ = 0.517]. All the interaction effects were not only highly significant (*p* < 0.001), but also very strong (η^2^ > 0.39). Pairwise comparisons further indicated that the mean scores of the three variables significantly increased from baseline to 3 months as well as from 3 to 6 months in the intervention group, while there were no pairs of time points where the mean scores were significantly different from one another in the control group (see Figs. 3, 4, 5). These results demonstrate that improvements on the treatment outcomes only occurred for the participants in the intervention group.

**Table 4:** Results of repeated measures ANOVA on effects of intervention on treatment outcomes for group, time, and group × time interaction

	Intervention group (N = 19)			Control group (N = 21)			Group effects		Time effects		Group × time effects	
	Mean ± SD			Mean ± SD								
	Baseline	3 months	6 months	Baseline	3 months	6 months	*p* value	Partial η2	*p* value	Partial η2	*p* value	Partial η2
AASE†	31.01 ± 12.51^a^	53.26 ± 9.99^b^	61.79 ± 10.84^c^	44.52 ± 16.59	42.24 ± 13.45	46.52 ± 17.70	**0.004**	**0.201**	0.074	0.068	** < 0.001**	**0.391**
	a < b***, b < c***			No significant pairwise differences								
EFI-K	70.53 ± 28.49^a^	99.21 ± 32.47^b^	120.32 ± 35.61^c^	75.38 ± 24.05	73.81 ± 22.95	74.29 ± 25.65	**0.012**	**0.154**	** < 0.001**	**0.525**	** < 0.001**	**0.548**
	a < b***, b < c***			No significant pairwise differences								
SAS‡	66.05 ± 13.44^a^	107.89 ± 20.65^b^	120.05 ± 24.90^c^	83.90 ± 21.65	85.90 ± 20.19	81.76 ± 20.76	**0.003**	**0.213**	** < 0.001**	**0.312**	** < 0.001**	**0.517**
	a < b***, b < c***			No significant pairwise differences								

Bold indicate significant *p* values and effect size, given as partial eta^2^, with medium to large effect (> 0.09)

*AASE* alcohol abstinence self-efficacy, *EFI-K* Enright Forgiveness Inventory-Korea, *SAS* Spirituality Assessment Scale

^†^Controlling for baseline spirituality

^‡^Controlling for baseline self-efficacy

^***^p < .001, Bonferroni

## Discussion

As the modified TC for women with AUD was never offered in an outpatient setting in South Korea, this study is positioned as a pilot trial. Two main findings were revealed in this pilot trial, which may be used to inform the design of a future definitive trial. First, the TC-oriented intensive day treatment program was feasible and well-accepted in the population of Korean women with AUD. Second, the 6-month treatment was significantly associated with increases in alcohol abstinence self-efficacy, forgiveness, and spirituality.

### Feasibility of the day treatment program

Seventy-nine percent of the participants in the intervention group successfully completed the treatment, demonstrating the feasibility of providing a TC-oriented day treatment program to women with AUD in South Korea. Completion rates are important in that they correlate with the post-treatment chances of recovery [36]. Yet, TCs have historically suffered from high drop-out rates [8]. The completion rate for our 6-month intensive treatment far exceeds those previously reported in the studies of TC-based substance abuse outpatient programs where retention rates ranged from 34 to 51% [13, 37, 38]. It should be noted, however, that the populations of the previous studies were not exactly the same as the population of the current study in that they included participants who were not only addicted to alcohol but also to other substances. The differences in retention rates may be ascribed to the differences in the substances to which these populations were addicted.

### Intervention effects on alcohol abstinence self-efficacy, forgiveness, and spirituality

Significant improvement in each of the three psychological outcomes (i.e., abstinence self-efficacy, forgiveness, and spirituality) was observed for the intervention group from baseline to treatment completion. Participants’ levels in these measures for the control group did not change over time. The findings suggest that alcohol abstinence self-efficacy, forgiveness, and spirituality may be cultivated over the course of a TC-oriented outpatient treatment program.

Several factors may have contributed to the therapeutic effect of the day treatment. First, the core elements of the traditional TC were retained in our treatment approach. TCs were originally designed to promote recovery going beyond mere abstinence from substance use. From the TC perspective, stable recovery is dependent on individual’s psychological growth and socialization [39]. It is possible that the TC principles and values that we adopted for our program such as the “community as method” strategy, social integration, a sense of responsibility for others, and mutual ‘self-help’ promoted spiritual growth and psychological well-being in participants [40]. Second, the treatment enhancements (e.g., counseling, cognitive behavioral therapy, case management) were culturally tailored to meet the unique needs of Korean women. That is, Korean women’s unique experiences, values, and beliefs were taken into account in the development of treatment content. For example, skills were taught to participants through role-playing on how to refuse invitations to the infamous Korean Hoesik (i.e., after-work gathering), where drinking is often mandatory and non-negotiable.

Another important factor was the incorporation of medical and mental health services into the treatments. Mental illnesses and substance use disorders often co-exist. In particular, research has found a strong comorbid association between PTSD and substance use disorders in women who had a history of physical and/or sexual trauma [41]. Indeed, more than two thirds of the study sample (n = 28) reported a lifetime history of either physical or sexual trauma/abuse: 70% for physical trauma/abuse and 20% for sexual trauma/abuse. During the trial, all participants were offered medical/mental health services (e.g., weekly visit to a psychiatrist) which may have addressed trauma and mental health symptomatology in them, resulting in promotion of individual wellness and recovery.

Although our results are very encouraging, the challenge of randomization is highlighted in this pilot study. Nine participants (22.5%) had to be allocated to the control group due to their personal needs. The most cited reasons for their inability to participate in the intervention were employment and family responsibilities. Participants who were employed full-time were afraid of losing their jobs if they attended the program since most employers would not allow them to take any time off from work. In fact, most women were very concerned about protecting their personal reputation and did not even want their employers to know about their need to receive treatment. Other participants were single mothers with children who preferred to stay at home to take care of their children. Although this challenge may be inherent in any addiction treatment study involving an intensive, structured daily schedule, it may be addressed by modifying the intervention in a way that allows for a more flexible treatment schedule or allows participants to bring their children to stay with them in the program.

Our findings may have implications for future treatment program design and policy. In South Korea alcoholism has been traditionally deemed as a men’s problem and almost all intervention programs have been targeting men. As we developed a women-only treatment program, we initially encountered a fair amount of skepticism. Even within the field, there were those who argued that women would not seek treatment, let alone stay in treatment long enough to achieve sobriety. Nevertheless, the findings from this pilot study provide support for the potential of a treatment program tailored to the specific needs of women as a stand-alone alcohol treatment. Indeed, some participants anecdotally commented that they found the women-only treatment environment much safer and more comfortable than the mixed-gender environment.

Another important point to note is our introduction of an intensive day treatment into the area of AUD treatment. Our findings build upon previous ones demonstrating that outpatient TCs could be as effective as residential TCs in promoting recovery among individuals with SUD [7]. This has implications for developing cost-effective treatment programs in South Korea. Residential TCs have been criticized often for their high running costs. With the rising cost of health care and budget constraints in South Korea, it may be in funding agencies’ interests to consider TC-based outpatient programs as a valuable alternative to traditional long-term residential treatment approaches.

### Limitations

Although impressive, the results of this study should be interpreted with caution given several limitations. First, the power calculation of the present study was based on the large effect size of one Korean TC study targeting a different population (i.e., men with SUD in a prison setting). Thus, the reported strong treatment effects may have been overestimated in consequence of the possibly underpowered study design. However, given that this is a pilot study and not an efficacy trial, the sample size may be small, but enough to allow us to examine the feasibility of an intervention. Another important limitation is that the participants were not randomly allocated to a treatment condition. Rather, allocation was sometimes determined based on the needs and preferences of participants (see the aforementioned reasons of some participants). There is a possibility that unmeasured baseline differences (e.g., motivation, treatment readiness) may have affected the study results such that less motivated participants preferred the control group. Therefore, the overall findings must be viewed in the context of potential selection bias. It should be noted, however, that true randomization may not be feasible in the field efficacy studies, nor may it be advisable, because self-selection factors could be predictors of treatment effectiveness [8]. Other limitations include the non-random sampling, the lack of fidelity monitoring, and the lack of an active control group.

### Conclusions

The current study points to the feasibility and acceptability of a TC-oriented intensive day treatment program for women with AUD living in South Korea. Our results also provide some initial evidence that the women-focused day treatment program may improve treatment outcomes not only in abstinence from alcohol use but also in psychological measures such as alcohol abstinence self-efficacy, forgiveness, and spirituality. The development of this intensive day treatment program is innovative in that it was the first women-focused and TC-based AUD treatment program in South Korea while incorporating an array of interventions designed to meet the unique needs of women. Future research is needed to assess long-term treatment effects as relapse rates tend to increase once individuals leave treatment [8]. At the same time, it would be worth exploring whether the duration of the current program could be even shorter considering the significant improvements observed in as early as 3 months from baseline. Lastly, the effect of selection factors (e.g., client motivation) on treatment outcomes needs to be investigated.

## Supplementary Information

**Additional file 1.** Complete dataset for all study variables.

## Data Availability

All data generated and/or analyzed during this study are included in the Additional file 1.
